# Implications of the variation in biological ^18^O natural abundance in body water to inform use of Bayesian methods for modelling total energy expenditure when using doubly labelled water

**DOI:** 10.1002/rcm.8291

**Published:** 2018-11-12

**Authors:** Priya A. Singh, Elise R. Orford, Kevin Donkers, Leslie J.C. Bluck, Michelle C. Venables

**Affiliations:** ^1^ Stable Isotope Facility MRC Elsie Widdowson Laboratory 120 Fulbourn Road Cambridge CB1 9NL UK

## Abstract

**Rationale:**

Variation in ^18^O natural abundance can lead to errors in the calculation of total energy expenditure (TEE) when using the doubly labelled water (DLW) method. The use of Bayesian statistics allows a distribution to be assigned to ^18^O natural abundance, thus allowing a best‐fit value to be used in the calculation. The aim of this study was to calculate within‐subject variation in ^18^O natural abundance and apply this to our original working model for TEE calculation.

**Methods:**

Urine samples from a cohort of 99 women, dosed with 50 g of 20% ^2^H_2_O, undertaking a 14‐day breast milk intake protocol, were analysed for ^18^O. The within‐subject variance was calculated and applied to a Bayesian model for the calculation of TEE in a separate cohort of 36 women. This cohort of 36 women had taken part in a DLW study and had been dosed with 80 mg/kg body weight ^2^H_2_O and 150 mg/kg body weight H_2_^18^O.

**Results:**

The average change in the δ^18^O value from the 99 women was 1.14‰ (0.77) [0.99, 1.29], with the average within‐subject ^18^O natural abundance variance being 0.13‰^2^ (0.25) [0.08, 0.18]. There were no significant differences in TEE (9745 (1414), 9804 (1460) and 9789 (1455) kJ/day, non‐Bayesian, Bluck Bayesian and modified Bayesian models, respectively) between methods.

**Conclusions:**

Our findings demonstrate that using a reduced natural variation in ^18^O as calculated from a population does not impact significantly on the calculation of TEE in our model. It may therefore be more conservative to allow a larger variance to account for individual extremes.

## 1 INTRODUCTION

The doubly labelled water (DLW) method is considered to be the “gold standard” for measuring free‐living total energy expenditure (TEE) in humans.^1^, ^2^ A bolus dose of ^2^H_2_^18^O is given and the dilution spaces and rate constants for ^2^H and ^18^O are calculated. Carbon dioxide (CO_2_) production is calculated from the difference in the elimination rates, with the ^2^H being lost as water, and the ^18^O as both water and CO_2_ via the bicarbonate‐water exchange in the blood. TEE is then estimated from CO_2_ production ( $R_{{\mathrm{CO}}_{2}}$) and the energy equivalent of CO_2_ using a respiratory quotient (RQ) or food quotient (FQ).^3^, ^4^

To calculate the elimination rates of ^2^H or ^18^O, it is first necessary to account for the natural abundance of isotope already present in the system. For the DLW method, it is typical to obtain a single pre‐dose sample, which can be plasma, saliva or urine, prior to the experiment, and take this as representative of the natural abundance throughout the measurement period. For ease of collection, this is commonly urine. The underlying assumption of the DLW method is that the natural abundances of both isotopes remain unchanged over the period of measurement. This is a consequence of two of the assumptions of the method; first that water leaves the body unfractionated and secondly that the intake is at the same isotopic enrichment as the body water.^5^, ^6^, ^7^ While these assumptions are known to be untrue,^7^, ^8^, ^9^ they are generally accepted, as it is not possible to directly measure natural abundance for either isotope during the measurement period. Therefore, either the natural abundance must be assumed to be unchanged or indirect methods must be used to overcome the likely variation.

To date there have been four such indirect methods: (1) dosing an individual to result in an optimal ratio between the two isotopes at the end‐period of the measurement. This has been shown to reduce the error due to natural variation by matching the slope of covariance between the isotopes. However, it is dependent on the size of the analytical error.^9^ Hence ideal ratios have varied between 6:1 and 12:1 delta values per mil of ^2^H to ^18^O. Whilst this takes into account the variation over the DLW period for the post‐dose samples, it still assumes that the measured pre‐dose value is a representative value in the calculation of TEE. (2) As an alternative to method (1), the use of a highly enriched DLW dose would mask the variation in natural abundance.^10^ However, this is an expensive method, which may be further complicated by concerns of accuracy in measuring such high enrichments and, as a result, it has not been utilised frequently.^11^ (3) Another proposal has been to use the natural variation in undosed participants to give a proxy of the natural variation within the dosed participant.^12^, ^13^ However, it has recently been shown that there is no inter‐individual correlation in time that would allow for this.^14^ (4) Interestingly, Berman et al^14^ did show that ^2^H, ^18^O and ^17^O were highly correlated and highlighted the potential for tracing the ^17^O isotope to account for variation in the former two isotopes within a DLW study period. Unfortunately, the technique used to manufacture ^18^O‐enriched water also enriches the ^17^O content, thus masking the ^17^O natural variation within a dosed individual, and rendering the possibility of using the ^17^O variation as a means of assessing the ^2^H and ^18^O variation currently unfeasible.

In the absence of any practical method to determine natural variation during a DLW experiment, here we investigate the use of modelling software to allow the natural abundance to vary from the measured value in the calculations to a best‐fit value over the period of measurement. This paper looks at calculating TEE using a Bayesian model in the free software WinBUGS.^15^ The WinBUGS software has been applied successfully to a wide range of physiological models, from gastric emptying^16^ to insulin sensitivity and the glucose minimal model.^17^

Bayesian methods allow for the incorporation of *a priori* knowledge (often referred to as priors) into the model and for uncertainty to be quantified; this is then modelled with the existing data to produce posterior probability distributions for the parameters of interest. For TEE there are a number of parameters for which prior knowledge is available, e.g. $R_{{\mathrm{CO}}_{2}}$ must be greater than zero and the fraction of body fat must lie between zero and one. The priors given may be informative or vague (otherwise known as non‐informative) depending on what is known about the probable distributions or how reliant the model is upon observed information. Within our model, *tauO* defines the variance for the distribution of ^18^O, assuming a normal distribution about the measured value. This allows uncertainty on the natural abundance which may then find a best‐fit value as determined by all the parameters described within the model. The use of Bayesian modelling can not only increase the likelihood of a successful result, for example in cases where the usual indicator of data quality, the space ratio (the ratio between the dilution spaces of hydrogen and oxygen, generally deemed acceptable between 1.015 and 1.060^18^), is outside the bounds of acceptability, but, by allowing uncertainty on the measurements, it can also account for the natural variation and this may then lead to a more confident determination of TEE.

Our original working model^19^ used a vague prior for the distribution of ^18^O, *tauO*. The aim of the present study was to quantify the natural abundance variation in ^18^O, and incorporate this into Bayesian modelling to allow a better determination of TEE. Within‐subject variation in ^18^O enrichment was calculated in 99 UK women (a cohort from the Diet and Nutrition Study of Infants and Young Children^20^) and used to modify our Bayesian model for DLW. Finally, both models were used to calculate TEE in an independent cohort of 36 UK women (National Diet and Nutrition Survey 2003 (NDNS)^21^).

## 2 EXPERIMENTAL

### 2.1 Participants

Participant data used for this study came from two previous cohorts. The first cohort was 99 UK women (Table 1) originally recruited as part of a breast milk intake study for the Diet and Nutrition Survey of Infants and Young Children (DNSIYC). The second cohort was 36 UK women (Table 1) who had previously taken part in DLW experiments as part of the National Diet and Nutrition Survey, 2003 (NDNS). All were informed of the purpose and nature of the studies and the potential risks involved, after which their written informed consent was given. The protocols were approved by the Cambridgeshire 4 Research Ethics Committee, Cambridge, UK, and the South Thames Multi‐centre Research Ethics Committee, London, UK, respectively.

**Table 1:** Subject characteristics for DNSIYC and NDNS cohorts

Variable	DNSIYC^a^		NDNS	
	Mean (SD)	95% CI	Mean (SD)	95% CI
Age (years)	33 (5)	[31,34]	43^b^ (13)	[38,48]
Weight (kg)	67.6 (11.9)	[65.2, 69.9]	69.0 (12.8)	[64.7, 73.4]
Height (m)	1.63 (0.07)	[1.62, 1.65]	1.63 (0.07)	[1.61, 1.66]
BMI (kg/m^2^)	25.3 (4.4)	[24.5, 26.2]	26.2 (6.0)	[24.1, 28.2]
k_H_ (day^−1^)	0.10 (0.02)	[0.10, 0.11]	0.09^b^ (0.02)	[0.09, 0.10]
N_H_ (moles)	1830 (290)	[1772, 1888]	1786 (210)	[1714, 1858]

k_H_, hydrogen rate constant; N_H_, hydrogen pool space.

a The women in the DNSIYC cohort were on average 11 ± 3 months post‐partum.

b Signifies a significant difference between cohorts.

### 2.2 General design

Within‐subject variance in ^18^O, over a 14‐day period, was calculated from the DNSIYC cohort and used to modify the Bluck Bayesian model for TEE determination. Data from the NDNS cohort was then used to calculate TEE using three methods: the method of Coward,^22^ (non‐Bayesian), Bluck Bayesian and modified Bayesian models.

### 2.3 Stable isotope analysis

Samples from the DNSIYC cohort, in which the women had been dosed prior to the start of a 14‐day urine sample collection period with 50 g of 20% ^2^H_2_O (CK Isotopes Ltd, Ibstock, UK), were analysed for ^18^O enrichment using the CO_2_ equilibration method of Roether.^23^ Briefly, 0.5 mL of sample was transferred into 12‐mL vials (Labco Ltd, Lampeter, UK), flush‐filled with 5% CO_2_ in N_2_ gas and equilibrated overnight whilst agitated on rotators (Stuart, Bibby Scientific). The headspace of the samples was then analysed using a continuous flow isotope ratio mass spectrometer (AP2003, Analytical Precision, Northwich, UK) alongside secondary reference standards previously calibrated against the primary international standards Vienna‐Standard Mean Ocean Water (vSMOW) and Vienna‐Standard Light Antarctic Precipitate (vSLAP) (International Atomic Energy Agency, Vienna, Austria). Sample enrichments were corrected for interference according to Craig^24^ and are expressed relative to vSMOW.

The NDNS cohort were dosed prior to the start of a 10‐day urine sample collection period with 80 mg/kg body weight ^2^H_2_O and 150 mg/kg body weight H_2_^18^O. Urine samples from the NDNS cohort were analysed for both ^2^H enrichment and ^18^O enrichment. ^2^H was measured using the reduction over uranium method^25^ (Aqua‐SIRA, VG Isogas, Middlewich, UK). ^18^O was measured using the CO_2_ equilibration method of Roether^23^ using the AP2003 continuous flow isotope ratio mass spectrometer as described for DNSIYC.

### 2.4 Calculations

All data considered in this paper are expressed in ‰ with respect to Vienna Standard Mean Ocean Water (vSMOW) on the delta scale:

$$\delta =\left(\left(\frac{R_{\text{samp}}}{R_{\mathrm{std}}}\right)-1\right)$$

where *R*_samp_ is the ^18^O/^16^O or ^2^H/^1^H ratio of the sample, and *R*_std_ is the corresponding ratio in vSMOW. Analytical precisions are better than ±0.12‰ for δ^18^O for the AP2003 and ± 1.5‰ for δ^2^H for the Aqua‐SIRA.

### 2.5 Total energy expenditure

Rate constants and dilution spaces are calculated from the slopes and intercepts of the log‐transformed data, with the rate of CO_2_ production, $R_{{\mathrm{CO}}_{2}}$ given by:^7^

$$R_{CO_{2}}=\frac{k_{O}N_{O}-k_{H}N_{H}-27.3\left(f_{2}-f_{1}\right)}{2f_{3}+1.1\left(f_{2}-f_{1}\right)}$$

where *k* and *N* refer to the rate constant and dilution space, respectively, with subscripts to indicate the isotope. The fractionation factors *f*_1_, *f*_2_, and *f*_3_ are given as 0.941, 0.991 and 1.037, respectively.

$R_{{\mathrm{CO}}_{2}}$ was converted into TEE using the following equation,^4^ with RQ assumed to be 0.85:

$$\mathrm{TEE}\;\left(\mathrm{kJ}.{\mathrm{day}}^{-1}\right)=22.4\times \left(\frac{15.48}{\mathrm{RQ}}+5.55\right)R_{{\mathrm{CO}}_{2}}\left(\mathrm{mol}/\mathrm{day}\right)$$

### 2.6 Bayesian modelling

A Bayesian model, based on the method of Coward, was written for WinBUGS.^19^ Parameter priors were assigned to the following: CO_2_ production rate, $R_{{\mathrm{CO}}_{2}}$; space ratio, *S*; water turnover, *R*_W_; and fraction of body fat, *F*. The priors were vague with the following distributions given: for $R_{{\mathrm{CO}}_{2}}$, a uniform distribution between 0 and 100 mol/day; for *R*_W_, a uniform distribution between 0 and 1000 mol/day; and, for *F*, a uniform distribution between 0 and 1. However, the prior *S* was given to be informative and assigned a normal distribution with a mean of 1.035 and standard deviation of 0.01.

The pool sizes and rate constants for H and O were described in terms of $\;R_{{\mathrm{CO}}_{2}}$, *S*, *R*_W_, *F* and body weight, with these described in the kinetic calculations for first‐order disappearance.

The within‐subject variance as calculated from the DNSIYC cohort was used to modify the basal ^18^O variation given in the Bluck Bayesian model;^19^ this is described by the parameter *tauO*, where:

$$\mathrm{tau}=\frac{1}{\text{variance}}$$

The parameter *tauO* was given the value of 4 in the Bluck Bayesian model.

The Bayesian modelling was completed using WinBUGS^15^ with 50,000 iterations in the Markov chain, the first 4000 being discarded as burn in. The total run time was 334 s and 341 s for the Bluck and modified models, respectively, on a 64‐bit standard desktop workstation (Dell Computers Ltd, Bracknell, UK) with 4GB RAM, and an Intel i5 processor, running Windows 7 (Microsoft Corp., Redmond, WA, USA).

### 2.7 Statistical analysis

The primary outcome measurement was the total energy expenditure (TEE) determined using the non‐Bayesian, Bluck Bayesian and modified Bayesian models. Secondary outcome measurements were *S* and $R_{{\mathrm{CO}}_{2}}$ (mol/day). Data analysis was performed using IBM SPSS Statistics for Windows, version 22.0 (IBM Corp., Armonk, NY, USA). The data are presented as means and standard deviation with 95% confidence intervals, and were checked for normality using the Kolmogorov–Smirnov test. To compare potential differences in the TEE calculated using the three methods, a one‐way repeated measures ANOVA was conducted. Agreement between the two Bayesian models was assessed using Bland–Altman plots with significance assessed using Student's t‐test. The level of significance was set at *P* <0.05.

## 3 RESULTS

### 3.1 Participants

The women from both the DNSIYC and the NDNS cohorts matched for all variables except for age. It can be seen that the women from the NDNS cohort were significantly older than those from the DNSIYC cohort (Table 1). The calculated variable of *N*_H_ was not significantly different between cohorts; however, *k*_H_ was 10% higher in the DNSIYC than in the NDNS cohort.

### 3.2 Natural abundance variation and calculation of TEE

**Figure 1:**
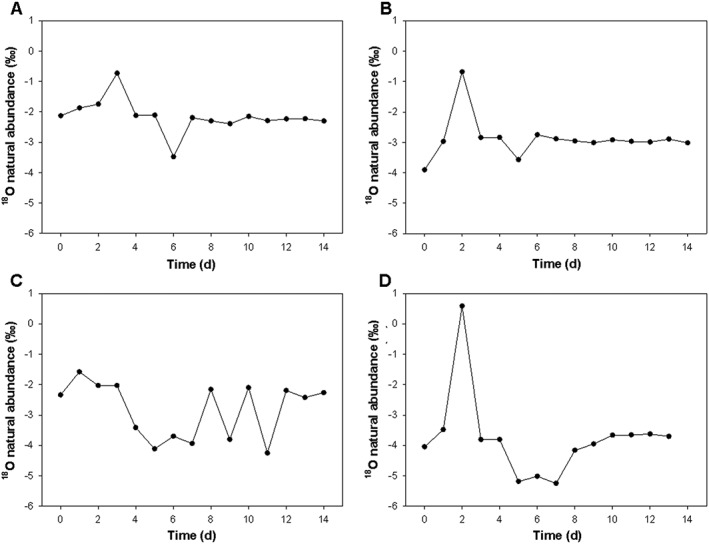
Urine ^18^O natural abundance across 14 days in three representative women from the DNSIYC cohort (A, B, C) with D displaying the individual with the greatest range

Figure 1 presents the natural abundance *δ* ^18^O values relative to SMOW for four individuals from the DNSIYC cohort over the 14‐day collection period. The three individuals (A, B and C) are representative of the cohort, while D is the individual with the greatest range. It can be seen that while the natural abundance may remain stable over several days, it is not consistent over the whole measurement period. Participant D undergoes considerable variation, fluctuating by 5.83 delta values (max – min). The average change in delta values (max – min) from the 99 women who formed the DNSIYC cohort was 1.14‰ (0.77) [0.99, 1.29], with the average within‐subject ^18^O natural abundance variance being 0.13‰^2^ (0.25) [0.08, 0.18].

**Figure 2:**
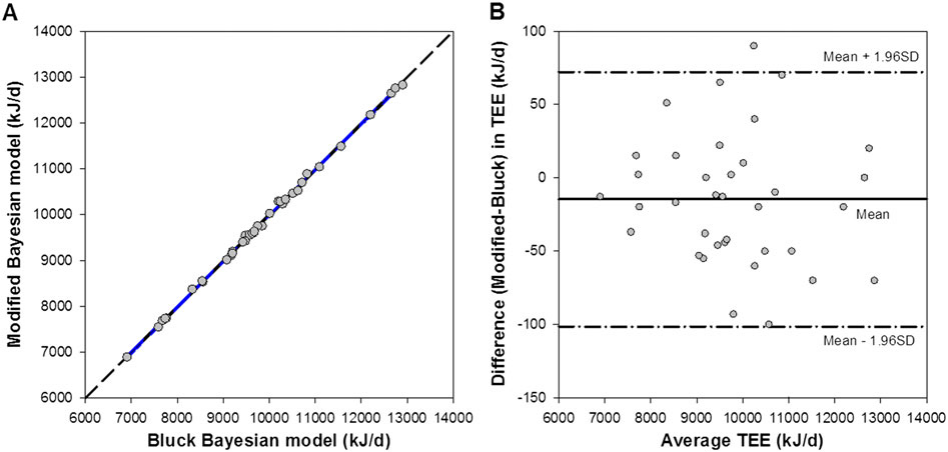
Pearson's correlation (A) and Bland–Altman (B) comparisons between Bluck and modified Bayesian methods for estimating total energy expenditure. For the Bland–Altman comparison, the solid line represents the mean difference between the two methods and the dashed lines the 95% limits of agreement (−103, 78 kJ/day) [Color figure can be viewed at http://wileyonlinelibrary.com]

The calculated natural abundance variance in ^18^O of 0.13 delta value squared replaced the variance and therefore *tauO* in the Bluck Bayesian model of TEE. The value of *tauO* for the Bluck Bayesian model was set to 4 and the modified value was set to 7.6. There were no significant differences in *S*, $R_{{\mathrm{CO}}_{2}}$ or TEE when calculated using each of the three methods (Table 2). There is a significant correlation between the two Bayesian models (Figure 2A, r^2^ = 1.000, *p* <0.05) and when comparing the agreement between the two Bayesian models using Bland–Altman analysis it can be seen that the modified Bayesian model had a negligible mean negative bias in TEE of 15 kJ/day (44) [−30, 0] (Figure 2B). There was no significant difference in TEE between the Bluck (9804 (1460) kJ/d) and modified (9789 (1455) kJ/d) Bayesian models; t(35) = 1.99, *p* = 0.053.

**Table 2:** Space ratio, $R_{{\mathrm{CO}}_{2}}$ and TEE for non‐Bayesian, Bluck Bayesian and modified Bayesian methods

Variable	Non‐Bayesian^a^		Bluck Bayesian^b^		Modified Bayesian	
	Mean (SD)	95% CI	Mean (SD)	95% CI	Mean (SD)	95% CI
S	1.037 (0.012)	[1.033, 1.041]	1.036 (0.006)	[1.034, 1.039]	1.037 (0.007)	[1.034, 1.039]
$R_{{\mathrm{CO}}_{2}}$	18.3 (2.7)	[17.4, 19.2]	18.4 (2.7)	[17.5, 19.4]	18.4 (2.7)	[17.5, 19.3]
TEE	9745 (1414)	[9267, 10224]	9804 (1460)	[9310, 10298]	9789 (1455)	[9297, 10282]

a Non‐Bayesian data is RQ fixed.

b Bluck Bayesian;^18^ S, space ratio; $R_{{\mathrm{CO}}_{2}}$, rate of carbon dioxide production (mol/day); TEE, total energy expenditure (kJ/day).

There was a significant difference between Bayesian models in levels of uncertainty of both $R_{{\mathrm{CO}}_{2}}$ and TEE. The uncertainty associated with $R_{{\mathrm{CO}}_{2}}$ was greater using the Bluck Bayesian model (7.22 (1.70)) than with the modified Bayesian model (6.00 (1.42)), t(35) = 25.03, *p* <0.05. As TEE is derived directly from $\;R_{{\mathrm{CO}}_{2}}$, the differences in uncertainty associated with TEE are identical to those of $R_{{\mathrm{CO}}_{2}}$.

## 4 DISCUSSION

The aim of the present study was to quantify natural abundance variation in ^18^O within a cohort of UK women, and incorporate this into our working Bayesian model to allow for a more robust determination of total energy expenditure.

The observed data shows that there are no differences between TEE for our NDNS cohort when calculated using either the Bluck or the modified Bayesian model. This would suggest that the Bluck Bayesian model has sufficient ability to allow for ^18^O variation in the model, so restricting *tauO* is unnecessary.

Typically, in non‐Bayesian methods of calculating TEE, the largest proportion of the error of the TEE estimate comes from natural abundance variation.^26^ It can be seen from our reported NDNS data that, when TEE is calculated using non‐Bayesian methods, the total error is 4.77 ± 1.29% as calculated according to Ritz et al;^26^ of this, the error arising from natural abundance variation is 4.36 ± 1.22%. This total error is comparable with that found in other studies.^26^, ^27^ It is calculated using regression statistics on the isotope enrichments and their products and ratios to calculate internal precision and, in addition, makes assumptions regarding the associated error of the single pre‐dose used.

The error analysis of the WinBUGS model is calculated differently and is not directly comparable with the non‐Bayesian error. It is instead based on the posterior distributions (levels of uncertainty) of the reparametrised Coward model for TEE. From the WinBUGS model, the posterior distributions of $R_{{\mathrm{CO}}_{2}}$ and therefore TEE result in levels of uncertainty of 7.22 ± 1.70% and 6.00 ± 1.42% for the Bluck and modified Bayesian models, respectively. This difference is significant and it is apparent that the altered ^18^O distribution (*tauO*) impacts upon the associated levels of uncertainty of $R_{{\mathrm{CO}}_{2}}$ and therefore of TEE. A narrower ^18^O distribution provides narrower posterior distributions for $R_{{\mathrm{CO}}_{2}}$ and TEE, and therefore reduces the uncertainty as it gives greater weight to the observed data.

The narrower ^18^O distribution reflects the smaller within‐subject variance calculated from our DNSIYC cohort, 0.13 delta value squared, rather than the estimate used by Bluck of 0.25 delta value squared. However, problems may arise when the natural abundance value used in calculations occurs at an extreme for that individual. This can result in under‐ or over‐estimation of TEE.

It may be better to use the larger variance for the ^18^O distribution, as there is no significant effect on TEE itself and, despite the increase in uncertainty, this will still give a similar result. By allowing a greater variance and applying it to the measured pre‐dose there is a greater likelihood that this would cover changes in delta values across our population, and so result in a more robust estimation of TEE. However, this may not necessarily cover all extremes. Therefore, it is up to the researcher to decide whether is it better to use a greater distribution for ^18^O and accept a larger uncertainty for $R_{{\mathrm{CO}}_{2}}$ or to reduce the associated uncertainty for all with the possibility of losing data at the extremes.

It is common practice in non‐Bayesian methods to take a single pre‐dose sample prior to dosing an individual as this provides the minimum required information on natural abundance; however, it can be seen from our data that across a 14‐day measurement period the ^18^O enrichment can change by up to 5.83‰. Taking multiple pre‐dose samples per participant allows calculation of the error due to natural abundance contributions,^26^ whereas using only one pre‐dose sample means that the natural abundance contribution to error can only be assumed. However, it should be borne in mind that taking multiple pre‐dose samples increases the participant burden and the cost of the measurement. For large epidemiological studies the increased cost and scheduling of these additional samples within the study design must be factored in.

Where a single pre‐dose sample is taken it is then better to use Bayesian methodology. The use of Bayesian statistics allows the measured value of the single pre‐dose to vary about a given distribution and reduce uncertainty. If, however, multiple pre‐dose samples are taken the error can be better estimated and non‐Bayesian methods may be more suitable.

With our data (DNSIYC), the within‐subject ^18^O natural abundance was found to vary with an average range in delta values of 1.14‰ (0.77) which is similar to the 1.16‰ (0.43) measured by Berman et al.^14^ The greater standard deviation than that reported in Berman et al^14^ can be explained by the inclusion of several subjects where the range is more extreme, with a maximum range of 5.83‰. The cause of this increased range in ^18^O could be that the population was more geographically diverse. Participants in our study were recruited from across the UK, rather than from one US city.

A further key point to remember is that the ^18^O natural abundance does not fluctuate alone; the ^2^H isotope also fluctuates in what has been shown to be a covariant fashion.^14^ Due to the covariant behaviour, the error resulting from natural abundance changes is reduced to a potential ±5% difference in TEE (data not shown). This is a similar error to that quoted by Schoeller^27^ and Ritz et al.^26^

### 4.1 Limitations and future work

Both cohorts were subsets of nationally representative surveys; however, neither subset has been chosen to be nationally representative and as such may be biased geographically. Darling et al^28^ reported that the isotopic composition of the UK groundwater varies depending on location within the UK and, as water source does have an effect on the isotopic natural abundance of total body water, further work in this area would be of interest and could include analysis regarding natural abundance variation across the UK and even further afield.

Although we are aware that the two studies were spaced approximately 10 years apart, this seems unlikely to matter as the same instrument and methods were used for the ^18^O analysis.

The DNSIYC cohort are younger than the NDNS cohort and it has been reported that water turnover is affected by age in children.^29^ However, our own data in adults (unreported NDNS Y1 and 3^30^) show that water turnover increases with age from about 20 years to 50 years of age, and so we would expect that the NDNS cohort would have greater water turnover if it was not for the fact that the DNSIYC cohort were breast‐feeding. Levels of breast‐feeding varied considerably from almost none to exclusively breast‐feeding, as the average age of the infants was 11 months. We have previously observed an increased rate of water turnover in our laboratory for breast‐feeding women (unpublished data), which in turn may impact natural abundance ^18^O and ^2^H variation. It is possible, therefore, that the increased water turnover observed in the DNSIYC cohort would result in swifter and more visible changes in natural abundance variation; however, this remains to be investigated.

## 5 CONCLUSIONS

The application of Bayesian methods is a superior methodology to calculate total energy expenditure (TEE) when a single pre‐dose has been taken, due to the ability to assign probability distributions to the known parameters. We sought to calculate ^18^O variation in a population and amend the variation used in our previous work (the Bluck Bayesian model). However, it would appear that for the calculation of TEE using the doubly labelled water method, the Bluck Bayesian model has sufficient in‐built flexibility to compensate for the variation in ^18^O.
